# The elimination of fox rabies from Europe: determinants of success and lessons for the future

**DOI:** 10.1098/rstb.2012.0142

**Published:** 2013-08-05

**Authors:** Conrad M. Freuling, Katie Hampson, Thomas Selhorst, Ronald Schröder, Francois X. Meslin, Thomas C. Mettenleiter, Thomas Müller

**Affiliations:** 1 Institute of Molecular Biology, Friedrich-Loeffler-Institut, WHO Collaborating Centre for Rabies Surveillance and Research, 17493 Greifswald–Isle of Riems, Germany; 2 Boyd Orr Centre for Population and Ecosystem Health, Institute for Biodiversity, Animal Health and Comparative Medicine, Graham Kerr Building Medical, Veterinary and Life Sciences, University of Glasgow, Glasgow G12 8QQ, UK; 3 Institute of Epidemiology, Friedrich-Loeffler-Institut, 16868 Wusterhausen, Germany; 4 Neglected Zoonotic Diseases (NZD), Department of Neglected Tropical Diseases (NTD), Cluster HIV/AIDS, Malaria, Tuberculosis and Neglected Tropical Diseases (HTM), WHO Headquarters, Geneva, Switzerland

**Keywords:** fox rabies, oral rabies vaccination, elimination, influencing factors, efforts/expenses, endgame

## Abstract

Despite perceived challenges to controlling an infectious disease in wildlife, oral rabies vaccination (ORV) of foxes has proved a remarkably successful tool and a prime example of a sophisticated strategy to eliminate disease from wildlife reservoirs. During the past three decades, the implementation of ORV programmes in 24 countries has led to the elimination of fox-mediated rabies from vast areas of Western and Central Europe. In this study, we evaluated the efficiency of 22 European ORV programmes between 1978 and 2010. During this period an area of almost 1.9 million km² was targeted at least once with vaccine baits, with control taking between 5 and 26 years depending upon the country. We examined factors influencing effort required both to control and eliminate fox rabies as well as cost-related issues of these programmes. The proportion of land area ever affected by rabies and an index capturing the size and overlap of successive ORV campaigns were identified as factors having statistically significant effects on the number of campaigns required to both control and eliminate rabies. Repeat comprehensive campaigns that are wholly overlapping much more rapidly eliminate infection and are less costly in the long term. Disproportionally greater effort is required in the final phase of an ORV programme, with a median of 11 additional campaigns required to eliminate disease once incidence has been reduced by 90 per cent. If successive ORV campaigns span the entire affected area, rabies will be eliminated more rapidly than if campaigns are implemented in a less comprehensive manner, therefore reducing ORV expenditure in the longer term. These findings should help improve the planning and implementation of ORV programmes, and facilitate future decision-making by veterinary authorities and policy-makers.

## 1. Introduction

Vaccination programmes are one of the most effective means of controlling infectious diseases [1]. Smallpox and rinderpest have been eradicated through vaccination programmes in people and cattle, respectively [2,3], and other diseases have been eliminated from large parts of the globe as a result of concerted vaccination efforts. Yet, wildlife diseases that pose a threat to public health, livestock production or conservation are less likely to be considered as candidates for vaccination programmes owing to the difficulty and expense in the mass delivery of vaccines to wild animal populations. Indeed, the lack of a wildlife reservoir is considered a prerequisite for a disease to be considered eradicable [4]. However, with the development of oral vaccines and bait delivery systems, the elimination of diseases circulating in wildlife populations has become a tantalizing possibility. The pre-eminent example of vaccination of wildlife populations is the large-scale oral vaccination programmes that have eliminated fox rabies from Western Europe and greatly reduced incidence in Central Europe [5]. Here, we focus on the effectiveness of these programmes, and the factors underlying the critical transition from disease control to the ultimate goal of disease elimination.

Rabies is one of the oldest recognized zoonoses, and is, with the exception of the Antarctic, present worldwide. The causative agents are negative-strand RNA virus species (previously genotypes) of the *Lyssavirus* genus, family Rhabdoviridae of the Mononegavirales order [6]. Of all *Lyssavirus* species known to date, the prototypic rabies virus (RABV) is the most important being maintained by a diversity of abundant canid and viverrid hosts across the world, and Chiroptera in the Americas [7]. This invariably fatal disease is a major public health threat [8]. Although human cases are preventable by prompt administration of post-exposure prophylaxes, control and elimination is only feasible in reservoir populations, e.g. domestic dogs and foxes, amongst others [9].

Dogs were recognized as a source of rabies infection in Europe several centuries ago. Over the first half of the twentieth century, measures such as dog movement restriction and muzzling drastically reduced incidence and led to freedom from dog-mediated rabies in some parts of Europe [10]. With the development and application of mass parenteral vaccination, canine rabies was eliminated from the continent, persisting only in the European part of Turkey [11]. However, parallel to this success, rabies virus emerged in red foxes (*Vulpes vulpes*) south of the Kaliningrad region following a presumed spillover from domestic dogs. Within a few decades, the virus was circulating among foxes across much of Central and Western Europe [12].

Early counteractive control measures aimed exclusively at reducing fox numbers. These met with only limited success, and eventually were regarded as counterproductive as they disrupted the social system of foxes, thereby increasing contact rates and disease incidence [13]. Mass parenteral vaccination was not considered to be a viable alternative [12,13]. However, experiments in the 1970s showed that red foxes could be immunized against rabies by the oral route leading to the concept of oral rabies vaccination (ORV) [14]. In 1978, the first ORV field trial was conducted in Switzerland [15] and was followed by efforts in other European countries, e.g. Germany, France and Belgium [16]. Thanks to pioneering attempts in a few countries in Western Europe and financial support from the European Union (EU) [10], it soon became evident that ORV was a breakthrough for fox rabies control. National elimination programmes using ORV were successfully implemented, and for approved programmes, 50 per cent (since 2010, 75%) of the costs for vaccine baits and bait distribution were reimbursed by the EU [10,17]. The EU also promoted the implementation of ORV programmes in neighbouring non-EU countries by co-financing a 100 km deep vaccination belt along common borders. Currently, the EU supports ORV in the Russian region of Kaliningrad, the Western Balkans and in northeastern neighbouring countries [18].

ORV programmes have been evaluated for several countries in Europe, including Belgium [19], Switzerland [20], France [21], the Czech Republic [22], Estonia [23,24], Germany [25,26] and Italy [27,28]. Prior to the successful elimination of rabies from countries in Western Europe, Stöhr & Meslin [29] compared the progress and setbacks of ORV. However, a comparative epidemiological analysis of ORV across Europe and its success in eliminating rabies has not been conducted. We, therefore, evaluate the efficiency of European ORV programmes in terms of controlling and eliminating fox rabies and we examine cost-related issues of these programmes. Our findings provide valuable insights into the effort and tactics required for rabies elimination, and guidance for decision making in future wildlife vaccination programmes by veterinary authorities, natural resource managers and policy-makers.

## 2. Material and methods

### (a) Study regions and oral rabies vaccination approach

The study regions encompassed countries from Western, Central and Eastern Europe that implemented ORV programmes between 1978 and 2010. Only countries in which more than four ORV campaigns were conducted during this period were considered for analysis (detailed in table 1). A standard ORV approach was applied that typically involved (i) implementation of ORV campaigns twice a year (spring and autumn), (ii) an average bait density of 20–25 baits km^−2^, (iii) aerial and manual distribution of vaccine baits, and (iv) a flight line distance of 500–2000 m in the case of aerial distribution. Occasionally, ORV campaigns slightly diverged from this protocol. In a few cases, ORV campaigns were only conducted once a year because of the specific epidemiological situation (Italy, 1984) or additional campaigns were conducted either in summer or in winter (France, Germany 2005, Italy 2009) or at short intervals (Germany, 2005; table 1). During the observation period a total of seven different SAD (Street Alabama Dufferin) derived oral rabies virus vaccines were used, including SAD Berne [15,23], SAD B19 [26,28,30], SAG2 [23,31,32], SAD P5/88, SAG1, SAD VA1 [26], Vnukovo32, and one recombinant vaccine, i.e. V-RG [33,34]. It is assumed that the efficacy of the vaccines used is comparable as requirements of the European Pharmacopoeia had to be met for these vaccines to be licensed.

**Table 1.** Data on the design of ORV campaigns conducted in different European countries.

country	year ORV began	cases at start of ORV^a^	cases in 2010	campaigns until elimination or end of 2010 (+)	campaigns until control^a^ (permanent control^b^ if different) or end of 2010 (+)	area of territory (km^2^)	border length with endemic areas (km)	vaccinated area (km^2^)	proportion of territory vaccinated	area index until elimination	area index until control (permanent, if different)	estimated costs (euros)
Austria	1986	1387	eliminated in 2006	42	20	78 527	1822	78 626	1	0.19	0.24	29 152 563
Belgium	1986	342	eliminated in 1999	29	14 (25)	28 582	471	12 329	0.43	0.41	0.38 (0.43)	2 987 759
Bulgaria	2009	58	6	4+	4+	105 510	1022	59 186	0.56	1	1	29 936 008
Czech Republic	1989	1712	eliminated in 2002	42	20 (24)	73 644	1433	73 644	1	0.59	0.57 (0.58)	10 287 231
Estonia	2004	314	eliminated in 2009	11	7	43 693	480	41 767	0.96	0.86	0.77	142 057 754
France	1986	2465	eliminated in 1998	25	17	514 550	1008	147 484	0.29	0.22	0.24	1 553 178
Germany	1983	10 484	eliminated in 2006	62	32	333 440	2242	293 290	0.88	0.19	0.23	1 564 850
Hungary	1992	892	11	37+	27	87 225	1495	87 225	1	0.5	0.44	11 067 650
Italy (1)	1984	354	eliminated in 1986	3	3	285 802	591	33 776	0.12	0.3	0.3	1 113 805
Italy (2)	1993	82	eliminated in 1995	6	6	285 802	n.a.	4544	0.02	0.48	0.48	4 205 706
Italy (3)	2009	68	209	4+	4+	285 802	n.a.	32 486	0.11	0.56	0.56	10 880 899
Latvia (1)	1999	169	n.a.	10+	10+	64 635	n.a.	60 978	0.94	0.83	0.83	12 978 745
Latvia (2)	2005	421	16	11+	11	64 635	n.a.	60 978	0.94	0.86	0.86	2 768 802
Lithuania (1)	1995	80	n.a.	11+	11+	61 011	n.a.	60 927	1	0.8	0.8	85 274 480
Lithuania (2)	2006	2232	33	10+	6	61 011	n.a.	60 927	1	1	1	998 959
Luxembourg	1986	137	eliminated in 1999	27	23	2419	231	2419	1	1	1	106 185
Poland	1993	2645	151	36+	24	291 133	2200	291 812	1	0.74	0.61	3 454 894
Russia (Kaliningrad)	2007	26	43	4+	4+	15 125	437	12 703	0.84	1	1	248 627
Slovakia	1993	489	eliminated in 2006	28	28	45 804	1160	45 804	1	0.73	0.73	22 961 998
Slovenia	1989	761	12	39+	13 (37)	19 167	815	19 047	0.99	0.71	0.47 (0.7)	17 006 552
Switzerland	1978	1054	eliminated in 1996	37	19 (35)	38 875	889	18 665	0.48	0.19	0.16 (0.19)	6 788 657
Ukraine	2007	2929	1862	6+	6+	558 161	1926	413 046	0.74	0.59	0.59	1 246 714

^a^Control is achieved when rabies cases dropped below 10% of the number of cases at the start of ORV.

^b^Permanent control is when cases dropped below 10% of the number of cases at the start of ORV and stayed below this until elimination or until the end of the observation period.

### (b) Data collection

As part of the terms of reference as a WHO Collaborating Centre for Rabies Surveillance and Research, national rabies surveillance data were collected and evaluated. Rabies cases presented here were laboratory confirmed using standard diagnostic techniques [35]. Records originated from regular official submissions from veterinary authorities of European countries to the WHO Rabies Bulletin Europe database established at the Friedrich–Loeffler-Institut (FLI), where rabies cases in different species are summarized for regions on a quarterly basis [36]. Surveillance was generally passive, based on submission of specimens by veterinarians, hunters, wildlife managers and the general public. International recommendations for rabies surveillance vary but suggest that either at least four or eight foxes per 100 km^2^ be tested annually [8,37,38]. Additionally, specimens obtained for monitoring of ORV campaigns were also examined. However, this surveillance, consisting of random sampling of foxes from the regular hunting bag (not suspect for rabies), revealed only a very small number of additional cases to those obtained through passive means.

For each country, annual animal rabies cases (not including bat rabies) from the year of the first implementation of ORV campaigns until the year of the last wildlife-mediated rabies case (rabies-free countries) or the year 2010 (rabies-endemic countries) were used for analysis. Additionally, for each country, data related to the ORV programmes were requested, including the size of individual vaccination areas, the timing of vaccination campaigns, bait density, mode of bait distribution and oral rabies virus vaccine strains used. For each vaccination campaign, e.g. in spring, in summer, in autumn or in winter, the size and location of vaccination areas was either requested as shape files or, if not available, as scanned maps. In the latter case, vaccination areas were digitized and converted into a GIS database (ArcGIS, Esri Inc., Redlands, CA, USA), as previously described [39].

### (c) Data analysis

We used a Cox-proportional hazard model [40] to investigate factors influencing the effort (number of campaigns) required to control and ultimately eliminate fox rabies through ORV. We used the number of ORV campaigns required for control, permanent control and elimination as the baseline hazard function, which was right censored for ORV programmes that had not achieved these endpoints by 2010. In this study, elimination was defined as a reduction of cases to zero. The fact that this situation has to be maintained for a minimum of 2 years after the last detected rabies case for a country to be officially recognized as rabies-free was not considered here. We defined control as a relative reduction in annual rabies cases by more than 90 per cent compared with initial endemic levels prior to implementation of ORV. We differentiated between control, when incidence is reduced by at least 90 per cent, but may subsequently increase, and permanent control, whereupon the 90 per cent reduction in rabies cases is maintained.

We considered the following variables as potential factors influencing the baseline hazard of effort required to control and eliminate fox rabies from a country: the size of the country (in km^2^); the area and proportion of the territory that was ever vaccinated (area given in km^2^); the length of borders to neighbouring rabies-infected (endemic) areas (in km); initial rabies incidence in the year prior to the implementation of ORV, expressed both as the number of detected cases and the number of detected cases per square kilometre; years of ORV experience prior to each country starting an ORV programme; and an index that captures the contiguity and overlap of consecutive ORV campaigns carried out in a country ([41]; table 2). Areas were log transformed, and proportions logit transformed, prior to statistical analysis. The best fitting model was selected using a stepwise algorithm that minimizes the Akaike information criterion.

**Table 2.** Variables in the statistical analysis of factors affecting the control and elimination of fox rabies using ORV.

variable	definition
initial cases, *N,* and initial cases per square km, *N*_km^2^_	the annual number of cases detected in the year when ORV began in a country, also expressed in terms of the number of cases detected per square kilometre (*N/vA*_max_)
vaccinated area, *V*_max_	the maximum area (km²) where vaccine baits were distributed, corresponding to the rabies endemic area
area of the territory, *A*	the total area (km²) of the country under consideration
proportion of the territory ever affected, *P*	the maximum proportion of the territory ever vaccinated (*P = V/A*)
border length, *B*	the length of the common boundary (km) between the area vaccinated in the focal country and the area vaccinated in neighbouring countries
area index, *I (I*_C100_, *I*_C90_ and *I*_C90p_)	an index capturing the mean spatial overlap and completeness of consecutive ORV campaigns in a country. The mean index was calculated from the start of ORV until the last detected case, *I_C_*_100_; until initial numbers of cases had been reduced by 90%, *I*_C__90_ (but may have subsequently increased); and until this reduction was maintained permanently, *I_C_*_90*p*_; see equation (2.1) and text
years of ORV experience, Y	number of years since ORV first began in Europe

For any fox rabies endemic area covered at least once with vaccine baits during the course of an ORV programme, we assumed that the rabies-affected area corresponded to the maximum area ever vaccinated in that country (*V*_max_ in km^2^). Vaccination areas were mostly bounded by administrative borderlines or natural barriers. The mean area index, *I*, is a metric designed to capture the comprehensiveness of ORV programmes in terms of the extent of spatial overlap of consecutive ORV campaigns (*d*) and the proportion of the endemic area covered, for the duration of the programme. The index is calculated from the area and overlap of consecutive campaigns *t* (*t* > 1) according to the following equations:2.1
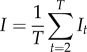
2.2
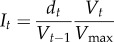
with V*_t_*, the size of the vaccinated area at campaign *t*, and *d_t_* is the intersection of successive campaigns *V_t_* and *V_t−1_*_._ The index varies from zero to one, from campaigns that are entirely non-overlapping to those that entirely overlap and are equal in size to the maximum area ever vaccinated [41]. If ORV campaigns were interrupted for more than one year, subsequent ORV campaigns were considered new ORV programmes and the area index was re-calculated (table 1).

Three models were setup with the number of campaigns required for control and elimination as dependent variables. Mean area indices were calculated for the corresponding duration of ORV programmes until control, permanent control, and elimination were achieved (*I*_C90_, *I*_C90p_ and *I*_C100_). Correlations between possible explanatory variables were examined using the Spearman rank correlation coefficient [42] and only uncorrelated variables were included in the analysis. The proportional hazards assumption for a Cox regression model fit was tested as described elsewhere [43,44].

Additional strategic variables relating to the design and implementation of ORV, e.g. number of vaccine baits, bait density or the modes of bait distribution (hand/aerial) were not considered in this analysis, because this information was not available for all countries or changed over time.

For the majority of European countries included in this study, direct and/or indirect expenditure on ORV programmes was not available. However, cumulative vaccination area over time can be used as a surrogate for financial expenditure on ORV following methods previously described by Selhorst & Schlüter [45]. In their analysis, it was shown that total expenditure on ORV is primarily owing to purchase of vaccine baits (market-based price) followed by costs of bait distribution [26,45]. Both costs are determined largely by the area vaccinated during each campaign, therefore the cumulative area vaccinated was considered a reasonable approximation of ORV programme expenditure. Total direct ORV programme costs (*c*, in euros) were approximated using the number of baits distributed per square kilometre (*b*) and the cumulative area vaccinated (*a,* in km^2^), as follows: *c = a*(0.82 *b* + 2.01) [30]. We assume 20 baits km^−2^ (*b* = 20), which is the standard bait density for oral vaccination of foxes in Europe, corresponding to 18.41 euros per square kilometre vaccinated.

We used a linear mixed effects model to determine the relationship between annual incidence in each county (*N_t_*) and approximate cumulative expenditure starting from the year of ORV implementation until the year the last rabies case was detected. Therefore the analysis was limited to countries that eliminated rabies. The model was fitted using restricted maximum likelihood, with country as a random effect (see the electronic supplementary material, figure S2) and the coefficient *λ* estimated as a fixed effect. The exponential decline in rabies incidence with cumulative vaccination effort was modelled since annual rabies incidence was log_e_ transformed. Using this relationship (equation (2.3)) we estimated the approximate financial expenditure *c*_1/*x*_ required to reduce initial rabies incidence at the start of ORV to *N_0_/x*.2.3
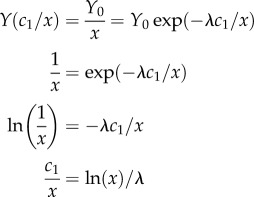
All analyses were performed using the ‘R’ programming language [46].

## 3. Results

**Figure 1.**
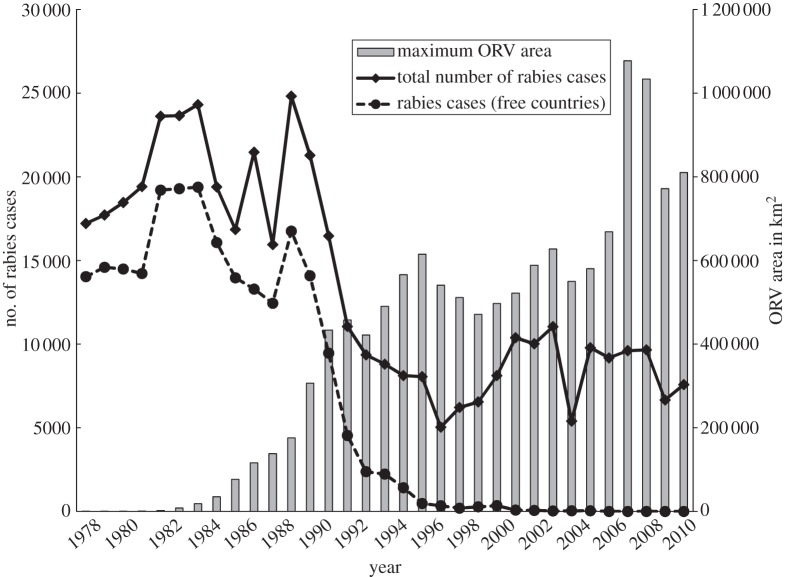
Reported annual rabies cases in Europe and extent of ORV programmes. The dashed line shows cases in countries which eliminated rabies (see table 1) and the solid line shows the total number of cases for all countries in Europe. Cases in bats and humans are excluded. The maximum area covered by oral rabies vaccination is shown in km².

**Figure 2.**
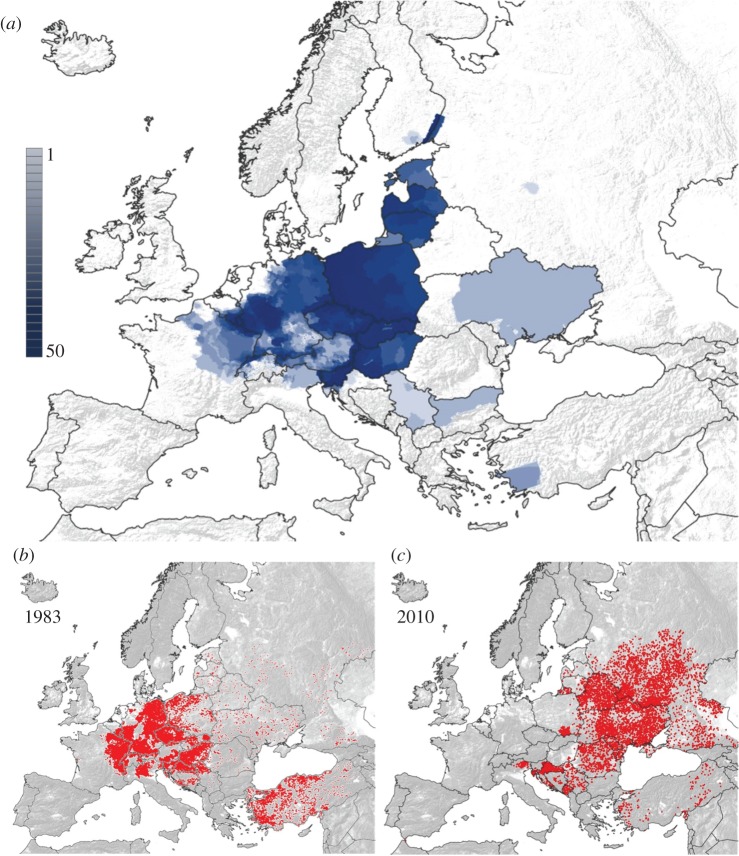
ORV effort and rabies cases. (*a*) Spatial extent of ORV area and the total number of ORV campaigns conducted in each country between 1978 and 2010. Reported rabies cases in (*b*) 1983 and (*c*) 2010.

During the past three decades, 24 European countries implemented ORV programmes on their territories. Since 1978 the overall size of the area under vaccination in Europe steadily increased to 614 773 km^2^ in 1996 as countries in Western and Central Europe began to implement ORV programmes. From 1997 until 2006, the area under vaccination remained relatively constant. Since 2000 ORV programmes in several countries have been discontinued following successful rabies elimination, while programmes have been initiated in countries in Eastern Europe (table 1). The total area under concurrent vaccination eventually peaked in 2007 at 1 077 370 km^2^ (figure 1). The total area ever covered at least once with vaccine baits between 1978 and 2010 encompassed 1.92 million km^2^. The spatial extent and frequency of ORV campaigns varied considerably, both within countries and at a regional level, with the total number of campaigns each country conducted between 1978 and 2010 ranging from one to 50 (figure 2*a*). In 12 countries ORV did not encompass the entire territory (figure 2*a*).

As a result of the ongoing implementation of ORV, the number of rabies cases reported annually in Europe steadily declined from 17 202 in 1978 to 7581 in 2010 with intermediate peaks in 1984 and 1989 (figure 1; electronic supplementary material, figure S1). During this period nine countries successfully eliminated fox rabies from their territory (figure 2*b,c* and table 1; electronic supplementary material, figure S1). The World Organization for Animal Health (OIE) requires that no case be detected for a two-year period before countries can be officially declared free from rabies [47]. Finland and the Netherlands achieved rabies-free status in 1991 [48], Switzerland in 1998 [20], Belgium and Luxembourg in 2001 [48], the Czech Republic in 2004 [22] and Germany and Austria in 2008 [26,49]. An imported dog rabies case with limited secondary transmission caused France to lose its rabies-free status in 2008 which it had achieved in 2000, but rabies freedom was regained in 2010 [10]. Italy became rabies-free in 1997, although fox rabies re-emerged on two subsequent occasions, most recently in 2008 (table 1). Additionally, Slovakia reported its last case in 2006 and could be regarded as rabies-free, although it has not been officially declared rabies-free according to OIE standards. Other countries now close to eliminating rabies include Hungary where the last reported case was in 2010 and Lithuania, Estonia and Italy where only single cases were reported in 2011 (www.who-rabies-bulletin.org).

The situation was very different amongst countries prior to the implementation of their ORV programmes. For instance, initial incidence ranged from 10 484 detected cases in Germany to just 26 in Kaliningrad Oblast, Russia (table 1). Much of this variation was attributable to the land area of countries with endemic rabies, as evident in the significant positive correlation between cases detected and territory size (Spearman's rank correlation, 0.43). However, there was still a large range in the number of cases detected per square kilometre, varying from 0.05 cases km^−2^ (Luxembourg) to less than 0.001 cases km^−2^ (Bulgaria). Several significant correlations were detected between other potential explanatory variables (see the electronic supplementary material, table S1). Correlated variables were therefore excluded from further analysis, leaving the area index, the proportion of the territory ever affected (*P*), the maximum area vaccinated (*V*_max_), and relative rabies incidence (number of cases detected per square kilometre, 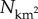) as possible explanatory variables for the analysis of effort required to control and eliminate rabies.

**Figure 3.**
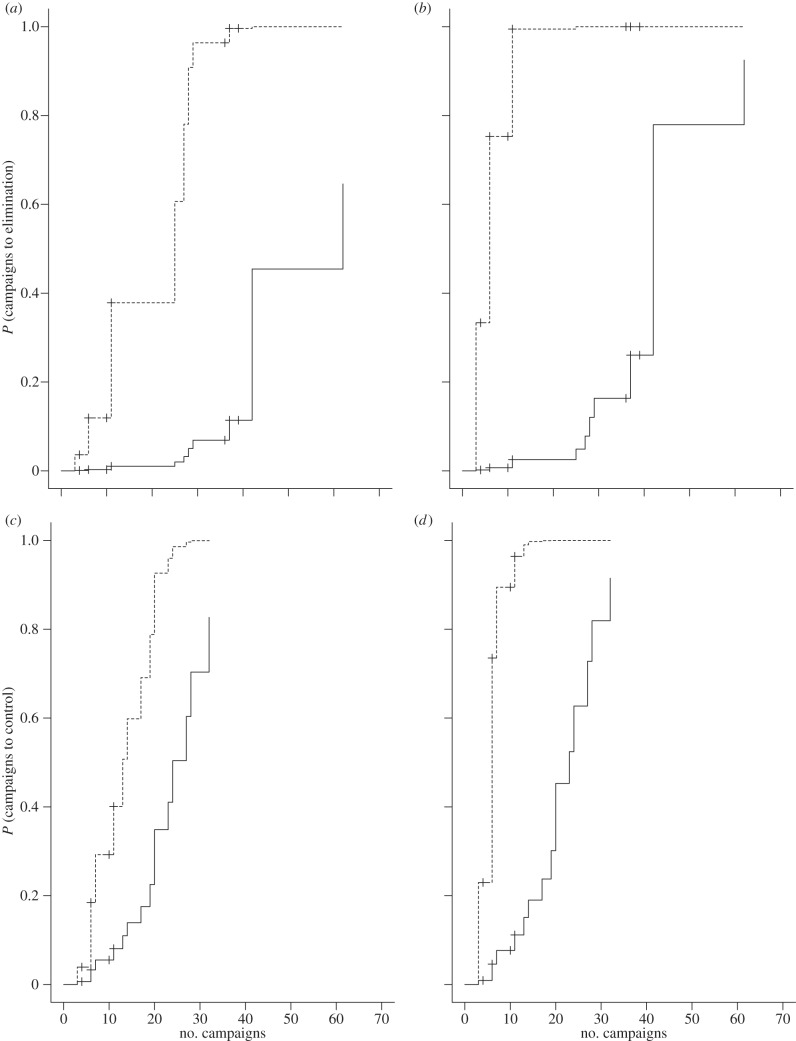
Factors influencing the efficiency of ORV programmes in the control and elimination of rabies. To illustrate the influence of the area index (*I*) and of the proportion of the territory ever affected (*P*) on numbers of campaigns to (*c*,*d*) control and (*a*,*b*) eliminate rabies, the continuous variables *I* and *P* are set to low and high values within the range of observed values. In (*a*,*c*) *I* is set to 0.2 (solid line) and 1.0 (dashed line) while *P* is set to 0.95 (median). In (*b*,*d*) *P* is set to 0.02 (dashed line) and 1.0 (solid line) while *I* is set to 0.65 (median). All curves are generated using parameter estimates obtained from the statistical analysis together with values for the influential parameter as described above. Survival curves are plotted as cumulative events, f(*y*) = 1 − *y*, and hash marks denote censored points.

Control of rabies to less than 10 per cent of initial endemic levels took a median number of 17 successive ORV campaigns, but ranged from three to 62 depending on the country. An additional four campaigns were required to achieve permanent control, and an additional 11 campaigns were required to eliminate rabies (median of 28 campaigns to achieve elimination versus 21 for permanent control and 17 for control). Following stepwise model selection, the area index and the proportion of a territory affected were found to have significant effects upon the number of campaigns required for control, permanent control and elimination (table 3 and figure 3). For countries that implemented consistently overlapping campaigns covering most of the affected area and therefore with a mean area index close to one, fewer campaigns were required in comparison with countries that implemented relatively incomplete and non-overlapping campaigns (for elimination: with *P* = 0.95 and *I*_C100_ = 1, a median of 25 campaigns were required, versus 62 with *I*_C100_ = 0.2, figure 3*a*; for control: with *I*_C__90_ equal to 1.0 and 0.2, 13 and 24 campaigns were required, respectively, figure 3*c*). In contrast, the greater proportion of the territory ever affected, the more ORV campaigns were necessary (for elimination: 42 versus six campaigns for *P* = 1.0 versus 0.02, with *I*_C100_ set to 0.65, figure 3*b*; for control: 23 versus six campaigns, figure 3*d*). These results suggest that an increase of one in the log-odds (logit) of the proportion of the territory affected decreased the hazard of elimination by 55 per cent and an increase of one in the log-odds of the area index more than doubled the hazard of elimination (217%), whereas the impacts on the hazard of control and permanent control were similar but slightly smaller. As initial numbers of cases detected per square kilometre increased, the number of campaigns required for permanent control also increased (table 3). For all the models, the Cox-Snell Pseudo *R*^2^ values were relatively low (*R^2^ =* 0.28 for control, *R^2^ =* 0.37 for permanent control, *R^2^ =* 0.48 for elimination, table 3).

**Table 3.** Factors influencing the efficiency of ORV programmes in the control and elimination of rabies. *I*, area index calculated until control, permanent control and elimination; *P*, proportion of territory ever affected; 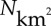, number of detected cases per square kilometre at the start of ORV; events, the number of countries which achieved rabies control, permanent control or elimination during 1978–2010.

Cox proportional hazard model	variable	transformation	hazard ratio (HR)	95% CIs	significance (*p*)	*n*	events	pseudo *R*^2^ (max. possible)^a^
control (90% reduction in rabies cases)	*P*	logit	0.61	0.42–0.88	0.008	22	16	0.278 (0.943)
	*I*_C90_	logit	1.439	0.97–2.13	0.069			
permanent control (maintained 90% reduction)	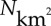		2.593 × 10^−20^	8.5 × 10^−39^−0.08	0.038	22	16	0.372 (0.943)
	*P*	logit	0.588	0.40–0.86	0.007			
	*I*_C90_	logit	1.86	1.01–3.40	0.045			
elimination	*P*	logit	0.4541	0.27–0.76	0.003	22	11	0.411 (0.854)
	*I*_C100_	logit	2.1679	1.11–4.23	0.023			

^a^Cox–Snell pseudo *R*^2^ value.

**Figure 4.**
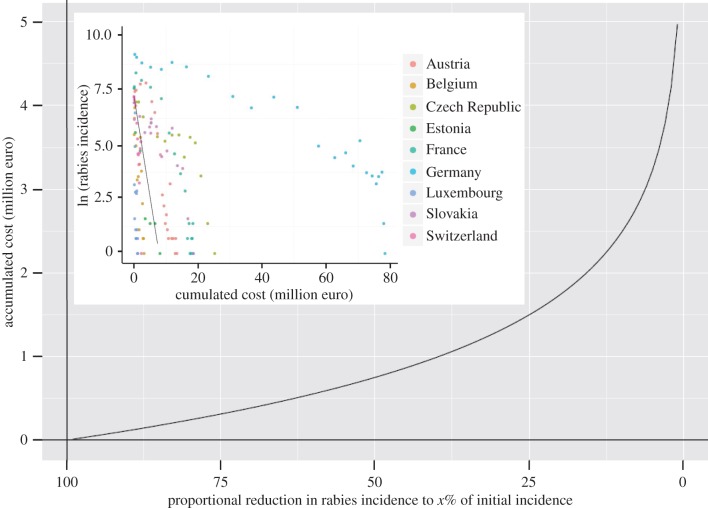
Estimated effort required to reduce rabies incidence. Accumulated financial expenditure (in euro) versus the proportional reduction in rabies cases from initial endemic levels. The inset shows the linear regression of log_e_ transformed annual rabies incidence (*y*-axis) against cumulative costs (*x*-axis) in million euros. A mixed model with country as a random effect was used to estimate *λ* (in equation (2.3). The fixed effect coefficient *λ* (−0.926) was used in the modelled relationship of the exponential decline in rabies incidence with increasing cumulative expenditure on ORV according to equation (2.3).

The cumulative area vaccinated and therefore the approximate expenditure by different countries to eliminate rabies varied considerably. Poland and Germany were amongst those that spent most on ORV, whereas rabies elimination in Switzerland was relatively inexpensive (table 1). For countries that eliminated rabies, a significant linear relationship was found between cumulative area vaccinated and log_e_ rabies incidence (figure 4). Although the exponential decline in incidence with increasing expenditure was evident for all these countries, considerable variation was evident from the random effects coefficients (see the electronic supplementary material, figure S2). This pattern indicated that increasing cumulative expenditure resulted in progressively smaller reductions in rabies incidence until elimination was achieved, albeit with high variation between countries (see the electronic supplementary material, figure S2). Cumulative effort to reduce initial incidence by 50 per cent followed an almost linear increase, whereas disproportionally greater effort was required in the final phase of elimination (figure 4). The approximate expenditure required to reduce initial incidence (*N*_0_) to *N*_0_/*x* was estimated as: c_1/*x*_ = log_e_(*x*)/−0.9257 (see equation (2.3), figure 4).

## 4. Discussion

The elimination of fox rabies from Europe represents possibly the most extensive and successful example of a vaccination programme targeting wildlife to date. In recent decades substantial progress in rabies control has been made in many parts of the world. Mass dog vaccination eliminated canine rabies from Europe and much of the Americas [10,50], almost exclusively relying upon parenteral delivery. The development of ORV has made the elimination of disease from wildlife reservoirs a reality. Over a period of three decades, vaccine baits have been distributed across nearly 1.9 million km² of Europe, with nine previously endemic countries now being rabies-free (figures 1, 2 and table 1; electronic supplementary material, figure S1,). Previous evaluations of ORV in Europe have focused on individual countries [19,20,23,26–28], with only one descriptive study assessing territorial differences and factors contributing to ORV success, including vaccine strains, and bait density. The latter covered the period from 1978 until 1994 when ORV was considered a work in progress as no countries had yet achieved rabies freedom [29]. Here, we provide the first comparative analysis of the efficiency of ORV in Europe and factors influencing the effort required to control and ultimately eliminate rabies, focusing on initial epidemiological conditions (table 1), geography and vaccination strategy.

Of the 24 European countries that have implemented ORV programmes, Finland and Turkey were excluded from our analysis. Finland was rabies-free until 1988 when fox rabies was introduced to the southeast of the country, most likely by rabid wolves. The resulting outbreak, which predominately affected raccoon dogs, was contained within a restricted area and eliminated after three ORV campaigns [51]. Turkey is the only country in Europe where dog-mediated rabies remains a problem. Sustained rabies transmission in foxes occurred in the Aegean region of Turkey following a spillover from dogs. Three ORV campaigns were undertaken during the winter months between 2008 and 2010. Although these demonstrated the feasibility of wildlife rabies control by ORV in Turkey [52], the impacts are difficult to assess for the entire country because of persisting dog-mediated rabies [10].

Successive ORV campaigns led to relatively rapid reductions in rabies incidence (table 1 and figure 4), typically bringing disease under control (more than 90% reduction in detected cases) within 10 years, and in many cases less than five years. However, the final stages of elimination typically required an additional 10 or more campaigns before the last case was detected, with ORV needing to be maintained for a further 2 years without any detected cases for certification. Previous research suggested that rabies could be eliminated from a region within 10–12 campaigns, i.e. 5–6 years [41,53]. But countries that successfully eliminated rabies in Europe required substantially longer (figure 3), with a median of 28 campaigns (14 years, range: 5–26) to achieve elimination, and 17 for control. Favourable topographical features may have contributed to the swift elimination of rabies from some European regions after only a few ORV campaigns, such as mountain ranges in Switzerland [31], while a number of other factors may have prolonged progress to elimination.

The proportion of a territory ever affected by rabies and the area index both had a decisive influence on ORV success (see the electronic supplementary material, tables S1 and S3; figure 3). The area index captures the comprehensiveness of ORV in terms of the extent and spatial overlap of consecutive ORV campaigns [41]. Among the 22 countries area indices varied considerably from 0.006 to 1 (table 1). The closer the mean area index was to one, the fewer campaigns were needed to both control and eliminate rabies (figure 3). A high mean area index reflects sufficiently sustained vaccination coverage in the fox population to maintain herd immunity and thereby interrupt rabies transmission, despite high population turnover [13,41]. The finding that the proportion of the territory affected correlates with campaigns required to control and eliminate rabies may reflect more isolated areas more quickly becoming free from disease, with elimination more likely in smaller regions simply by chance [41]. Otherwise, modelling suggests that time to control or elimination should be independent of the size of the affected area, assuming the same ORV strategy was applied [53].

Speculation that countries with high incidence, e.g. Switzerland, Austria, Germany and Hungary (table 1) require greater effort to eliminate disease [29] was not corroborated, though countries with higher incidence per square kilometre required more ORV effort to achieve maintained reductions in rabies incidence. This may be indicative of a more enduring effect, as improvements in surveillance may have reduced the power of our analyses of control, because we used initial endemic levels as a baseline. Surveillance considerably improved in some countries across the continent over the last few decades, even as the disease was progressively eliminated (figure 2*b,c*). International recommendations have suggested sample sizes for wildlife rabies surveillance [8,37,38]. While useful for monitoring bait uptake, this approach is arbitrary and inappropriate for case detection, because only suspect animals should be examined [54]. Our analyses instead suggest that it is more important to ensure that the extent of surveillance is sufficiently widespread for case detection across the endemic area, to effectively define the area for ORV.

ORV campaigns are undoubtedly easier to organize and manage across smaller spatial scales. In large countries ORV is often hampered by management and structural deficiencies. For instance, federal structures in Germany and Poland likely caused difficulties in individual regions [25,26,55] prolonging elimination at a national level (table 1). Although ORV programmes should ideally cover the entire affected area [41], larger countries often started ORV in restricted areas owing to logistical challenges and resource availability [26,32,48]. This is illustrated by the example of Romania, where despite approval for reimbursement of ORV costs by the EU, no budget was allocated for some years delaying implementation (P. Demetriou 2012, personal communication). The Baltic countries did not vaccinate the entire affected area from the beginning [23], and initial ORV attempts in Latvia and Lithuania failed to efficiently control rabies and were interrupted owing to budget constraints (table 1). Hence, subsequent ORV did not benefit from previous efforts and resources were wasted.

The type of vaccine used has been reported to be key to ORV success [23,48], however regional assessments have failed to reveal any association [29]. We, therefore, assume negligible differences between vaccines used in this study since all met the international regulations for quality control. Unfortunately, strategic factors other than the area index, e.g. optimal timing of campaigns, proportion of aerial versus hand distribution, exact bait density and variation in flight line distances, could not be analysed because of incomplete data [56–58].

We did not find any effect of shared borders between countries on the number of campaigns required for control or elimination**.** Yet, numerous examples of incursions (France–Switzerland (1990), France–Belgium–Germany (1993), Italy–Slovenia–Austria (1993), Germany–Poland–Czech Republic (1995) and Italy–Slovenia (2008) [26,28,29,59]) demonstrate the risk posed from neighbouring endemic areas and the need for coordinated cross-border activities [21,23,26]. In 2012, fox-mediated rabies spread from the Former Yugoslavian Republic of Macedonia (FYROM) into Greece, a country which was previously rabies-free (www.who-rabies-bulletin.org), whereas insufficient cross-border cooperation between federal states was the main obstacle to elimination in Germany [26]. Our analysis only crudely captures shared border length for the entire observation period while the risk varies over time depending on the rabies situation in adjacent areas. A more comprehensive approach that accounts for the inherent spatiotemporal transmission dynamics at border regions is therefore merited in future.

Costs of ORV vary greatly across North America and Europe [23,26,60–62] and detailed cumulative costs (direct and indirect) can only be calculated on a country basis. Nevertheless, direct ORV costs are largely driven by the area vaccinated, allowing a rough approximation of investment required for proportional declines in incidence [45]. Despite substantial differences between countries, the greater proportion of a territory infected, the higher are the costs of control (table 1 and figures 3, 4). Besides the proportion of the territory affected, there are several other reasons why for example Poland and Germany are amongst those that spent most on ORV (table 1). In Germany, in the late 1990s, residual foci of infection persisted in the federal states of North-Rhine Westphalia, Saxony and Hesse, the latter leading to re-infection of adjacent areas in Baden-Württemberg and Rhineland-Palatinate, greatly prolonging rabies elimination [26]. Despite considerable progress in Poland, legal issues prevent the cessation of ORV in rabies-free regions, at great expense. An upsurge of rabies in the Malopolskie region in 2010 in Poland caused a similar setback to that faced by Germany. While the reasons for this remain speculative [55] it is likely that this area was somewhat neglected in ORV efforts. Further analyses are needed to elucidate country-based differences in the cost effectiveness of ORV.

Our analysis demonstrated a roughly exponential decline in rabies cases with cumulative ORV expenditure. Therefore although ORV efforts reduce incidence, more effort is needed for the same payoff during the endgame (figure 4). This result is in line with the findings from the Cox-proportional hazard model that once rabies has been brought under control, which on average requires 17 ORV campaigns, an additional 11 ORV campaigns are needed to progress from a 90 per cent reduction in incidence to elimination. A similar trend has been observed for other elimination programmes, e.g. polio and smallpox [63–65]. Modelling results corroborate this phenomenon of long-lasting low-level persistence of rabies within vaccinated populations, both in wildlife and in dogs [53,66]. Despite vaccination suppressing incidence, clusters of infection are able to persist and spread, even in areas with a high area index; high levels of coverage are needed for more rapid elimination [53], whereas even small pockets of low coverage can compromise success and considerably extend the time to elimination [66]. Rapid reductions in incidence led to overly optimistic projections of ORV success by veterinary authorities, and premature budget cuts and/or declaration of areas as being ‘rabies-free’, frequently followed by subsequent re-emergence [58]. It is, therefore, of utmost importance to consolidate available resources to ensure comprehensive coverage eliminates any residual foci and prevents escalating costs.

Most EU member states and a few countries bordering the EU have eliminated rabies, or are on target for elimination in the near future, while countries in Eastern Europe are in the early stages of control. The establishment of a ‘cordon sanitaire’ is required to prevent the return of rabies to these areas, which means that border countries will always remain in the ‘endgame’ [67]. The EU is promoting the establishment of a vaccination belt in rabies endemic non-EU countries by co-financing [18]. Although largely effective, these immune barriers are not entirely impermeable, as the 2008 incursion into Northern Italy from the Balkan peninsula beyond the vaccination zone in Slovenia showed [68]. But the emergence of fox rabies in Greece in 2012 could have been anticipated, given that no preventive vaccination belt had been established when cases were detected in neighbouring border areas in the FYROM. All European countries also need to maintain a high level of vigilance, as rabies could be introduced via illegally imported animals [69], and awareness often falls following a prolonged absence of disease and cessation of ORV will lead to a build up of susceptibles.

## 5. Conclusions

The sustained effort and enormous geographical extent of ORV (figures 1 and 2) that has led to the successful control and elimination of fox rabies from vast areas of Europe is unprecedented for a wildlife disease [10]. We show that the extent to which a territory is affected influences the time to elimination, with ORV campaigns implemented in a comprehensive sustained manner (high *I*) more rapidly bringing disease under control. The final phase of elimination is disproportionally the most costly (figure 4), with increasing ORV effort resulting in diminishing reductions in rabies cases. Once brought under control, almost as many ORV campaigns are needed again to eliminate infection and maintain vaccination for long enough to certify freedom from disease. Therefore, a concerted ORV strategy including common coordination of cross-border activities is essential to save costs, with enough resources retained for the elimination stage. Our results indicate that the most efficient ORV programmes should vaccinate fully across the extent of the affected area and should secure adequate funding for a sufficient period of time to enable sustained vaccination with a high area index [28]. Despite the initially high investment needed for this strategy, fewer campaigns will be required to eliminate rabies, resulting in cost savings (figure 3). In any case, funding should include campaigns for two additional years after the last rabies case [51]. While rapid early successes in disease control are encouraging, policy makers must be prepared for continued commitment for elimination, and not be tempted to prematurely discontinue elimination programmes, because the final mile requires the greatest investment.
